# Authors’ response: on the role of data, statistics and decisions in a pandemic

**DOI:** 10.1007/s10182-022-00460-w

**Published:** 2022-07-30

**Authors:** Beate Jahn, Sarah Friedrich, Joachim Behnke, Joachim Engel, Ursula Garczarek, Ralf Münnich, Markus Pauly, Adalbert Wilhelm, Olaf Wolkenhauer, Markus Zwick, Uwe Siebert, Tim Friede

**Affiliations:** 1 Institute of Public Health, Medical Decision Making and Health Technology Assessment, Department of Public Health, Health Services Research and Health Technology Assessment, UMIT – University for Health Sciences, Medical Informatics and Technology, Hall in Tirol, Austria; 2 Department of Medical Statistics, University Medical Center Göttingen, Göttingen, Germany; 3 Zeppelin University Friedrichshafen, Friedrichshafen, Germany; 4 Pädagogische Hochschule Ludwigsburg, Ludwigsburg, Germany; 5 Cytel Inc, 675, Massachusetts Avenue, Cambridge, MA 02139 USA; 6 Economic and Social Statistics, Trier University, Trier, Germany; 7 Department of Statistics, TU Dortmund University, Dortmund, Germany; 8 Psychology and Methods, Jacobs University Bremen, Bremen, Germany; 9 Department of Systems Biology & Bioinformatics, University of Rostock and Leibniz-Institute for Food Systems Biology, Technical University of Munich, Munchen, Germany; 10 Goethe University Frankfurt, Frankfurt, Germany; 11 Institute for Technology Assessment and Department of Radiology, Massachusetts General Hospital; Harvard Medical School, Boston, MA USA; 12 Center for Health Decision Science and Departments of Epidemiology and Health Policy & Management, Harvard T.H. Chan School of Public Health, Boston, MA USA; 13 Institute of Mathematics, University of Augsburg, Augsburg, Germany

We are grateful to Berger et al. (2022b); Contreras et al. (2022); Höhle (2022) and Radermacher (2022) for their insightful comments. Overall, it seems that we hit a nerve in the statistical community with our discussion of the role data and statistics played in the COVID-19 pandemic and continue to play in other crises. We do not attempt to address all the points mentioned in the commentaries but focus on some of the main themes that were raised by several discussants.

An important aspect that is particularly relevant for the special situation of a pandemic is time. There are several facets to this issue: First, availability of data and thus the choice of possible models change over the course of the pandemic. In our overview of modeling approaches, we also discussed the role data play in each of the different models. However, we did not explicitly stress that some data might not be available in the early stages of a pandemic, and thus, not all models may be applied. Contreras et al. (2022) refer to this as a learning process and highlight the fact that the Bayesian framework lends itself to summarizing the occurring information. As Höhle (2022) stresses, however, a lack of data does not justify the use of over-simplistic models, since the choice of statistical methods massively impacts the final results (Nicola et al. 2022). Similar arguments also hold for clinical trials, where time pressure should not be used as an excuse to drop standards such as randomization or sample size calculation. Otherwise, results may be dramatically biased (Friedrich and Friede 2020).

Second, timeliness and accuracy are often in conflict with one another. This applies to the context of data quality as well as to timely communication of scientific findings and results. While we discuss ‘ideal’ standards that should be followed, we also see the point of not always being able to adhere to them in the interest of time. However, we believe that it is important to strive to uphold these standards, while at the same time communicating clearly in which aspect they had to be dropped. Berger et al. (2022b) added some statistical concepts to the discussion. Firstly, they highlight the importance of natural experiments and provide an example of school closures (Berger et al. 2022a). Secondly, they discuss the role that network analyses can play, in particular in situations with sparse data (Fritz and Kauermann 2022).

In our paper, the main focus was on the COVID-19 pandemic. The processes described, however, also apply to other crises and are relevant to all decision processes, be they political, social or even personal in nature. In order to implement the suggestions made in our paper and enhanced by the discussants, we need a collaborative effort to establish statistical experts in relevant panels. Radermacher (2022) emphasizes the importance of strengthening the cooperation between method development and application. This comment is in line with discussions held in the session entitled ‘Mind the gap - Interplay between theory and practice’ at the recent DAGStat conference (DAGStat 2022). Especially in an evolving ‘big data world’, it is crucial to establish standards, not only for data collection but also for the appropriateness of statistical models and the communication of results.

We would like to thank the discussants once more for adding their thoughtful comments. We agree with Prof. Radermacher that the statistical community should be more pro-active in offering solutions to the society for challenges we face—not only with respect to the pandemic but also to other problems such as the climate change debate. We believe that professional and learned societies could play an active role in this, building upon lessons learned in the pandemic.
